# Pathological impact and medical applications of electromagnetic field on melanoma: A focused review

**DOI:** 10.3389/fonc.2022.857068

**Published:** 2022-07-22

**Authors:** Yunxiao Duan, Xiaowen Wu, Ziqi Gong, Qian Guo, Yan Kong

**Affiliations:** ^1^ Astronomy Department, Wellesley College, Wellesley, MA, United States; ^2^ Melanoma Department, Beijing Institution for Cancer Research, Beijing, China

**Keywords:** magnetic field, melanoma, metastasis, apoptosis, tumor impair, nanotechnology, medical physics

## Abstract

Electromagnetic Field (EMF) influences melanoma in various ways. EMF can be classified into extremely low-frequency electromagnetic field, low-frequency magnetic field, static moderate magnetic field, strong electromagnetic field, alternating magnetic field, and magnetic nanoparticles. Each type of EMF influences melanoma development differently, and the detailed influence of each specific type of EMF on melanoma is reviewed. Furthermore, EMF influences melanoma cell polarity and hence affects drug uptake. In this review, the impacts of EMF on the effectiveness of drugs used to treat melanoma are listed according to drug types, with detailed effects according to the types of EMF and specific melanoma cell lines. EMF also impacts clinical therapies of melanoma, including localized magnetic hyperthermia, focalized thermotherapy, proton radiation treatment, nanostructure heating magnetic hyperthermia, radiation therapy, Polycaprolactone-Fe_3_O_4_ fiber mat-based bandage, and optune therapy. Above all, EMF has huge potential in melanoma treatment.

## Introduction

Melanoma is the most incurable form of skin cancer, and its rates are rising faster than any other cancer that can be prevented until now (1). Several factors have been recognized as risk factors for melanoma, including exposure to ultraviolet radiation (UVR), gene mutation, and family history. In particular, UVR exposure is the major cause of melanoma. Recently, scientists discovered that UV-induced DNA damage and melanoma susceptibility are regulated by the nuclear receptor coactivator NCOA3. In melanoma cell lines and patient-derived xenografts, downregulation of NCOA3 expression, either through genetic silencing or small-molecule inhibition, markedly reduced melanoma proliferation (2). Since UVR is a significant impact factor for the pre-clinical and clinical treatments of melanoma, all of the contents below maintain UVR as a constant factor unless specifically mentioned otherwise.

Electromagnetic field (EMF) is a force field that has both electric and magnetic components. It is generated by the motion of electric charges and has a wide range of frequency and energy (3). EMF influences the processes of metabolism in human body and imposes various biological effects on cells through multiple mechanisms (4). Therefore, EMF intervention is a common and vital method to aid and strengthen the quality of medical care for cancers. For instance, EMF-programmed magnetic vortex nano-delivery system is effective for breast cancer (5). EMF is also helpful for treating glioblastoma multiforme. By modulating the expression of O^6^-Methylguanine-DNA Methyltransferase, Cyclin-D1, and p53, discontinuous and pulsed EMF exposure reduces temozolomide resistance in glioblastoma (6).

Because UV radiation belongs to a specific range of electromagnetic radiation, it is reasonable to investigate how EMFs of different frequencies and intensities influence melanoma. Besides, many other types of cancer are influenced by EMF, implying a correlation between EMF and melanoma. Hence, many scientists are experimenting with EMF to treat and mitigate melanoma, as well as researching on the pathogenesis of melanoma with EMF.

In this review, we will discuss how distinct types of EMF influence melanoma development, including extremely low-frequency electromagnetic field, low-frequency magnetic field, static moderate magnetic field, strong electromagnetic field, alternating magnetic field, and magnetic nanoparticles. EMFs are classified according to their intensities, dynamics (static or alternating), and expression (field or particle). In addition, we will investigate how EMF affects uptake for various types of anti-cancer drugs, and how EMF impacts clinical therapies for melanoma.

## EMF’s influence on melanoma development

To better compare the impact of various EMFs on melanoma development and treatment, all pre-clinical studies in this section are concluded in **Table 1**.

**Table 1:** Pre-clinical studies: the impact of different types of EMF on melanoma development. .

EMF	Specific Value/Type of EMF	Influence on Melanoma	Melanoma Cell Line	Reference
Extremely Low-Frequency Electromagnetic Field	7.83±0.3 Hz	Inhibit melanoma growth	B16-F10	(7)
	8-24 Hz	Promote planarian growth and restrict malignant cell proliferation	B16-B6	(8)
	Thomas-EMF (25 Hz:200 ms + 6 Hz:500 ms)	Reduce melanoma cell growth while promote calcium absorption	B16-BL6	(9)
		Reduce growth of malignant cell lines while not affect non-malignant cells	B16-BL6 MDA-MB-231 MCF-7 HeLa cells	(10)
		Alter cellular cAMP and stimulate ERK phosphorylation in melanoma	B16-BL6	(11)
	50 Hz	Increase anti-apoptotic protein BAG3 levels and induce tension in melanoma cells	M14	(12)
	60 Hz	Enhance melanin secretion and hence inhibit melanoma growth	B16-F10	(13)
Low-frequency Magnetic Field	2-5 nT	Inhibit development of tumor	B16-BL6	(14)
	1 μT	Inhibit melanoma growth	B16	(15)
	0.4 T	Reduce melanoma cell proliferation and metastasis and improve immunological function	B16-F10	(16)
Static Moderate Magnetic Field	217.3 ± 3.0 mT	Produce scaffold-free surface culture of melanoma cells	B16-F10	(17)
	230-250 mT	Reduce fibroblast attachment and subsequently inhibit melanoma growth	WI-38	(18)
	586 mT	Impair angiogenesis and growth of solid tumors	A-Mel-3	(19)
	0.7 T	Reduce expression and activity of antioxidant enzymes in melanoma cells	C32	(20)
Strong Electromagnetic Field	900 MHz	Stimulate clathrin-dependent endocytosis, detach melanoma cell membrane	B16-F10	(21)
	Millimeter-wave (30-300 GHz)	Induce apoptosis of melanoma cells	A375	(22)
Alternating Electromagnetic Field	5 – 350 kHz	Electroporate melanoma cells, and change the frequency of cellular movement	B16-F10	(23)
	366 kHz	Reduce tumor size in synergy of bi-magnetic nanoparticles	B16-F10	(24)
	835.25 kHz	Decipher intracellular events triggered by mild magnetic hyperthermia	B16-F10	(25)
	950 kHz	Assess apoptosis in response to magnetic hyperthermia	DX3	(26)
Magnetic Nanoparticles	Fe	Boost the expression of key immunological and cytotoxic molecules in melanoma cells	B16-F10	(27)
	Fe_3_O_4_	Enhance targeting of angiopoietin 2-small interfering RNA plasmid/chitosan in melanoma cells	A-375	(28)
	SCMIOPs	Cytotoxic for melanoma cells in a dose-dependent manner	A375 B164-A5	(29)
	NPrCAP/magnetite	Restrict T-cell receptor repertoire in tumor-draining lymphocytes	B16-F1	(30)
	Irondextran nanoparticles	Enhance T cell proliferation and repress melanoma growth	B16	(31)

### Extremely low-frequency electromagnetic field

Extremely low-frequency electromagnetic fields range from 1 Hz to 300 Hz (32). Many biological systems are influenced by exposure to extremely low-frequency electromagnetic fields (ELF-EMF); these effects are mostly linked to the ELF-EMF’s strength, duration, frequency, and pattern. Below we collected ELF-EMFs of frequencies from low to high and explained their impact on melanoma.

#### 7.83 ± 0.3 Hz

The development of melanoma is inhibited by exposure to square waves at 7.83 ± 0.3 Hz (sweep step 0.1 Hz) in the B16-F10 mouse model. After 48 hours of exposure to the square wave, *in vitro* tests revealed a decrease in B16-F10 cell growth and an increase in Ca^2+^ influx. Inhibition of voltage-gated L- and T-type Ca^2+^ channels also help to reduce Ca^2+^ inflow (33). For 24 and 48 hours, B16-F10 melanoma cells are exposed to a natural EMF resonance frequency (7.83 Hz) and a power line frequency. To quantitatively assess the viability of melanoma cells, the B16-F10 cells are also subjected to sweep frequencies in different sweep intervals. In comparison to the control group, the data shows a 17% inhibition rate at 7.83 Hz. Furthermore, small interval sweep frequencies result in a 26.4% inhibition rate, and inhibitory effects decline as frequency sweep intervals grow (7).

#### 8-24 Hz

Synergies are discovered between a physiologically structured electromagnetic field (8-24 Hz), which is known to promote planarian growth and restrict B16-B6 melanoma cell proliferation in culture, and three light-emitting diodes (LED)-generated visible wavelengths (blue, green, and red) on planarian regeneration and melanoma cell quantities. Five days of hourly exposures to either a physiologically patterned magnetic field, one of three wavelengths (3 kLux), or both treatments at the same time reveal that red light (680 nm), blue light (470 nm), or the magnetic field significantly aids planarian regeneration when compared to sham field exposed planarian. The impact is amplified by presenting both light and magnetic field conditions. Although blue and red light reduces the growth of malignant melanoma cells, the effect is not as strong as the magnetic field. Only when the blue light and magnetic field are shown together does the suppression improve. Green light (540 nm) exposure, on the other hand, has no effect on the planarian or melanoma cells, and green light combined with the magnetic field completely abolishes the effects of the magnetic field. This finding suggests that some hues have a positive adaption effect comparable to weak, biologically patterned frequency-modulated electromagnetic fields, and the two types of energy can sum or cancel synergistically (8).

#### Thomas-EMF

Thomas-EMF is an irregular frequency-modulated sequence that consists of a 3 millisecond (ms) waveform repeated at 25 Hz for the first 200 ms of presentation, followed by a gradual drop to 6 Hz for the last 500 ms. Thomas-EMF reduces cell growth while promoting calcium absorption. Thomas-EMF is made up of 849 points in a digital-to-analog file that powers solenoids and can be programmed to change the timing, strength, and duration. Setting the point duration to 3 ms results in a time-varying EMF pattern that starts at 25 Hz and decelerated to 6 Hz over the course of 2.5 seconds. After 5 days of exposing B16-BL6 cells to Thomas-EMF set at 3 ms for 1 hour per day, cell growth is reduced by 40%, whereas adjusting the point duration to 1, 2, 4, or 5 ms has no impact. Likewise, cells exposed to Thomas-EMF for 3 ms show a three-fold increase in calcium uptake after 1 hour, but the other timings have no influence. Cell growth is reduced by exposure to Thomas-EMF for as little as 15 minutes per day, while treatment for 1 hour per day is best. This matches the impact of Thomas-EMF on calcium uptake, which elevates after 15 minutes of exposure and peaks after 1 hour (9). Thomas-EMF exposure for 1 hour daily reduces the growth of malignant cell lines such as B16-BL6, MDA-MB-231, MCF-7, and HeLa cells, while no effect is observed on non-malignant cells. *In vivo*, B16-BL6 cells are implanted in syngeneic C57B mice and subjected to Thomas-EMF on a daily basis, resulting in smaller tumors than sham-treated controls. *In vitro* investigations reveal that exposing malignant cells to Thomas-EMF for more than 15 minutes increases Ca^2+^ influx, which is inhibited by inhibitors of voltage-gated T-type Ca^2+^ channels. The Thomas-EMF-dependent inhibition of cell growth is likewise inhibited by blocking Ca^2+^ uptake. Thomas-EMF exposure slows cell cycle progression, alters cyclin expression, and reduces cell proliferation. Yet Ca^2+^ influx and cell proliferation of non-malignant cells are not affected by Thomas-EMF (10). The adenosine 3’,5’-cyclic monophosphate (cAMP) and extracellular signal-regulated kinase (ERK) signaling pathways are involved in the Thomas-EMF-induced alterations in cell proliferation. Thomas-EMF transiently alters the amount of cellular cAMP and stimulates ERK phosphorylation in malignant cells. The capacity of Thomas-EMF to suppress cell proliferation is prevented by pharmacologic inhibitors (SQ22536) and activators (forskolin) of cAMP generation, while a mitogen-activated protein (MAP) kinase pathway inhibitor (PD98059) is able to partially block Thomas-EMF-dependent reduction of cell proliferation. In B16-BL6 cells, genetic modification of protein kinase A (PKA) changes the effect of Thomas-EMF on cell growth. Thomas-EMF also inhibits the proliferation of cells transfected with the constitutively active version of PKA (PKA-CA), which interferes with ERK phosphorylation (11).

#### 50 Hz

Exposure to high temperature, oxidants, and other stressful factors causes the expression of anti-apoptotic protein BAG3 up-regulated in melanoma cells. *In vitro* and in orthotopic xenografts, exposure to 50 Hz electromagnetic fields increases BAG3 levels in the human melanoma cell line M14. BAG3 protein levels increase significantly (P<0.01) when cultivated cells or xenografts are exposed for 6 hours or 4 weeks, respectively. By recognizing BAG3 protein as a marker of ELF-induced tension, this finding corroborates the stressful effect of ELF exposure in human cells (12).

#### 60 Hz

Melanin determines the color of skin, and a lack of melanin causes various hypopigmentation diseases, as well as increased ultraviolet B (UVB) sun damage to the skin. 60–75 Hz ELF-EMFs enhance melanin secretion, and cellular melanin concentration by stimulating the expression of tyrosinase and tyrosinase-related protein-1 (TRP-1) *via* inhibition of phosphorylation extracellular-regulated kinase (ERK), activation of cyclic adenosine monophosphate (cAMP) response element-binding protein (CREB), and microphthalmia-associated transcription factor (MITF) up-regulation in B16-F10 melanoma cells, while mitochondria activity, cell viability, and cell membrane condition remain unaltered. Specifically, 60 Hz ELF-EMFs inhibit ERK phosphorylation in B16-F10 melanoma cells (13).

### Low-frequency magnetic field

Beside using Hertz (Hz) as a measurement of frequency similar to ELF-EMFs, low-frequency magnetic field (LF-MF) also uses the unit of Tesla (T) to denote the flux density of the magnetic field (34). Below is a collection of LF-MFs in the order of increased magnetic flux density. In general, LF-MFs also inhibits melanoma cell growth.

#### 1-5 nT

The development of tumors is inhibited by exposure to spatial-temporal controlled electromagnetic when mice are exposed to LF-MF field combinations. In two blocks of trials separated by six months, C57B male mice are injected subcutaneously with B16-BL6 melanoma cells, tumor development is measured after the mice are exposed to the same time-varying electromagnetic field nightly for 3 hours in one of six spatial configurations or two control situations. Mice exposed to a field that rotates across all three spatial dimensions and all three planes every 2 seconds for 38 days do not develop tumors. The mice in the sham-field and reference control developed enormous tumors after 38 days. The strength of the field influences tumor growth: animals exposed to a modest intensity field (1–5 nT) develop smaller tumors than mice subjected to sham or stronger, high intensity (2–5 mT) fields. Tumor immunohistochemistry from mice exposed to varied intensity fields shows that disparities in leukocyte infiltration or vascularisation can lead to variations in the development of tumors (14).

#### 1 μT

Behavioral, physiological, and cellular activity are all affected by weak (1 μT) biologically structured magnetic fields. In one study, 12 temporal samples of the electroencephalographic abnormality and normal activity of a person whose proximity reliably impacts the brain activity of others are taken from quantitative electroencephalographic (QEEG) data. The QEEG data is digitized and given to B16 mouse melanoma cells as comparable magnetic field patterns. Compared to the other patterns derived from his QEEG or sham field exposures, only two of the patterns, both coming from the primary source (right temporal lobe) of the electroencephalographic (EEG) abnormality, reduce cell proliferation by one-third. This finding shows that melanoma cell development can be inhibited when the innate complexity of certain people’s EEG patterns is amplified correctly and applied as computer-generated magnetic fields in three spatial planes (15).

#### 0.4 T

Low-frequency magnetic field (LF-MF) reduces melanoma cell proliferation and metastasis while improving immunological function in tumor-bearing animals. After exposure to the LF-MF (0.4 T, 7.5 Hz), the proliferation of B16-F10 cells declines, the cell cycle is arrested, and chromatin breakdown is observed. Furthermore, in a melanoma metastasis mouse model, LF-MF increases animal survival and reduces melanoma growth. LF-MF also influences immune response by regulating immune cells and cytokine production (16).

### Static moderate magnetic field

The impact of a static magnetic field (SMF) is investigated on the growth of several human cell types. Exposure to SMF has a significant biological impact on some, but not all types of human cells, including melanoma.

#### 217.3 ± 3.0 mT

There is a scaffold-free surface culture of B16-F10 murine melanoma cells based on magnetic levitation in a static magnetic field of 217.3 ± 3.0 mT. Without the need for a supporting scaffold, multicellular spheroids may be grown in several three-dimensional (3D) culture methods. Magnetic levitation of B16-F10 cells that have eaten Fe_3_O_4_-containing fibroin microspheres results in a floating disk-shaped 3D culture. The melanoma disk is up to 19 mm in diameter and is between 80 and 100 μm thick. The 3D culture is densely packed with cells that are multiplying at a rate of μ = 0.015 h^-1^. Ki-67 (a nuclear protein) positive cells are made up around half of the cells, and no apoptotic or autophagic cells are seen. The proportion of propidium iodide-permeable cells is 8.5 ± 1.2%, which is primarily attributable to physical damage to the cell membrane induced by Fe_3_O_4_-containing microspheres in a high magnetic field. Due to an augmented population of pigmented cells in the 3D culture, melanin synthesis rises by a ratio of 3.0–3.7 (17).

#### 230-250 mT

In one study, SMF is created by putting two magnets on either side of a T25 flask that is oriented in opposing directions. Cell number is plotted at 18 hours, 4, 7, 11, and 14 days following seeding to create growth curves, with the 18-hour point serving as a measure of attachment efficiency. When compared to a sham-exposed control, SMF exposure dramatically reduces fibroblast attachment and subsequently inhibits growth. Human melanoma cells do not adhere to SMF; however, SMF does limit cell growth by 20% on day 7. During the first 18 hours after seeding, when cell attachment occurs, oxidant generation increases by 37% in WI-38 cells, which are exposed to SMF (230–250 mT). However, no increase in oxidant levels is found after a 5-day exposure (18).

#### 586 mT

SMF can impair angiogenesis and the growth of solid tumors. SMFs cause a reduction in blood flow in tumor arteries as well as platelet activity and adhesion. Experiments are carried out on Syrian Golden Hamsters with syngenic A-Mel-3 melanoma; animals are immobilized three days after tumor cell implantation and subjected to an SMF of 586 mT for three hours. Control animals without being exposed to SMF are immobilized for the same amount of time. Field effects on tumor angiogenesis and microcirculation are studied for 7 days using *in vivo* fluorescence microscopy. The tumor development is significantly slowed (by 30%) after exposure to SMFs. Additionally, histological examination reveals that tumors exposed to SMFs have greater peri- and intratumoral edema. When comparing SMF-exposed tumors to control tumors, microcirculatory metrics indicate a substantial reduction in functional vessel density, vessel diameters, and red blood cell velocity. Enhanced edema in response to SMF exposure implies increased tumor microvessel leakiness, which can improve medication absorption (19).

#### 0.7 T

Moderate-strength static magnetic field also influences melanoma cells (amelanotic C32 cell line) with co-exposure to chlorogenic acid. The melanoma cells are placed in special magnetic test chambers that create a 0.7 T magnetic field, quantitative reverse transcription-polymerase chain reaction (RT-qPCR) is used to examine the antioxidant enzymes at mRNA levels, and the activities of superoxide dismutase (SOD), glutathione peroxidase (GPx), and catalase (CAT) are assessed in the cell lysates. The chlorogenic acid (CGA) treatment alone (1 mmol/L) reduces the expression and activity of antioxidant enzymes compared to untreated cells, while the CGA in combination with SMF does not. The cytotoxicity of CGA (1 mmol/L) and its suppression of antioxidant enzymes imply that phenolic chemicals could be beneficial as a complementary pharmacological therapy for melanoma (20).

### Strong electromagnetic field

Strong electromagnetic field influences melanoma cell growth in a more powerful way. Below is a collection of strong EMFs in the order of increased frequencies.

#### 900 MHz

900 MHz modulated electromagnetic fields accelerate the clathrin-mediated endocytosis. B16-F10 murine melanoma cells are exposed to Lucifer Yellow (LY) and global system for mobiles (GSM)-EMF/electric pulses in the presence of drugs inhibiting the clathrin-/caveolin-dependent endocytosis. Experiments are carried out in a wire patch cell with a specific absorption rate (SAR) of 3.2 W/kg, a homogeneously distributed EMF field, and a regulated temperature (between 28.5 and 29.5 Celsius). Therefore, the observed increase in LY uptake could not be attributed to a temperature impact. This rise is reduced by chlorpromazine and ethanol, but not by Filipin. As a result, the strong EMF stimulates clathrin-dependent endocytosis, implying that the cellular process influenced by the modulated EMF includes vesicles that detach from the cell membrane, primarily clathrin-coated vesicles (21).

#### Millimeter-wave (30-300 GHz)

Millimeter-wave (MMW) induces apoptosis of human melanoma A375 cells. MMW exposure in cells is stimulated using electromagnetic field calculations and the specific absorption rate (SAR) is estimated. The best irradiation settings are established based on the homogeneity and intensity of SAR. MMW is administered to A375 cells for 15, 30, 60, or 90 minutes, with or without pre-treatment with the caspase-3 inhibitor AC-DEVD-fmk (10 mol/L) for 1 hour at 90 minutes before the exposure. MMW exposure substantially reduces cell viability in a time-dependent way in the cell counting kit-8 (CCK-8) test, doses of 15, 30, 60, and 90 minutes all result in significantly increased cell apoptosis, and caspases-3 expression is substantially enhanced. Pre-treatment of the cells with AC-DEVD-fmk considerably reduces the inhibitory impact of MMW on cell viability (22).

### Alternating electromagnetic field

Alternating electromagnetic field (AMF) has various impacts on the medical treatment of melanoma. Below is a collection of AMFs in the order of increased frequencies.

#### 5-350 kHz

Alternating magnetic field (AMF) is used in testing changes of cell electrical parameters induced by electroporation. Dielectrophoresis is utilized to differentiate electroporated and non-electroporated cells. When cells are electroporated, the electric field frequency at which they change the direction of their movement (the crossover frequency *f*_CO_) is more significant. Using a single shell model, the contribution of four electric and geometrical cell characteristics on the cell dielectrophoretic behavior is investigated. *f*_CO_ measurements are carried out on B16-F10 cells electroporated in a Mannitol solution (0.001 S/m) using rectangular or exponential pulses in the medium with conductivities of 0.001–0.09 S/m. The *f*_CO_ of control cells range from 2 to 105 kHz, while the *f*_CO_ of electroporated cells range from 5 to 350 kHz depending on the external medium conductivities. Electroporated cells’ *f*_CO_ become substantially greater than controls at external conductivities over 0.02 S/m. Although the role of membrane conductivity in explaining the observed *f*_CO_ shift toward higher values cannot be ruled out, the simulations show that cytosol conductivity fluctuation is likely to be the primary contributor to the dielectrophoretic behavior change (23).

#### 366 kHz

In one study, the effect of bi-magnetic nanoparticles (MNPs) coupled with external AMF exposure on the development of B16-F10 subcutaneous mouse melanomas is investigated. After intratumoral or intravenous injection, bi-magnetic Fe/Fe_3_O_4_ core/shell nanoparticles are developed for cancer targeting. Organic dopamine-oligoethylene glycol ligands shield its inorganic core from fast biocorrosion. The dopamine-oligoethylene glycol ligands have 4-tetracarboxyphenyl porphyrin (TCPP) units linked to them. With three short 10-minute AMF exposures (366 kHz), the magnetic hyperthermia results show that micromolar quantities of iron supplied within the modified core-shell Fe/Fe_3_O_4_ nanoparticles have a strong anti-tumor impact on murine B16-F10 melanoma. A decline in tumor development is perceived after injecting MNPs intravenously and exposing the mice to AMF for three days straight 24 hours later. These findings suggest that injecting surface modified MNPs into melanoma after exposure to AMF can reduce the tumor size in mouse model (24).

#### 835.25 kHz

AMF is also used to decipher intracellular events triggered by mild magnetic hyperthermia. Murine melanoma cell line B16-F10 are exposed to an AMF (835.25 kHz, 20.05 kA/m) or macroscopic heating after being treated with magnetic nanoparticles. The effects of therapies are evaluated at the molecular, cellular, and animal levels. In the absence of cell ablation or a global temperature increase, thermotolerance pathways are discovered in the system by comparing hsp70 gene expression after treatments. Hsp70 transcriptional activity can be utilized as a molecular thermometer to detect cell reaction to magnetic hyperthermia (25).

#### 950 kHz

AMF can help assess apoptosis in response to magnetic hyperthermia. AMF is applied to DX3 human melanoma cells that are loaded with citric-acid-coated iron-oxide nanoparticles. Time-varying magnetic fields of amplitude 6.6, 10.5, 12.0, 14.7, and 16.1 kA/m are applied at a constant frequency of 950 kHz for a total treatment period of 2 hours per sample. Fluorophores are used to monitor pathways *in vitro* in suspensions and *in situ* in monolayers to report on early-stage apoptosis and/or necrosis. The pace and degree of delayed-onset effects are related to the thermal-load-per-cell. Without any observable change in the local environment temperature, membranal internal-to-external lipid exchange precedes rupture and death by a few hours (the timeline changing cell-to-cell) under moderate loads (26).

### Magnetic nanoparticles

One particular kind of EMF is magnetic nanoparticle-induced EMF. It influences melanoma development with more accuracy than other types of electromagnetic fields.

#### Fe

There are also immunogenetic effects of low-dose magnetic nanoparticle hyperthermia (mNPH) and radiation in melanoma cells. To achieve the desired thermal dosage, B16-F10 melanoma cells containing magnetic nanoparticles (mNPs, 2.5 μg Fe/106 cells) are pelleted and subjected to an AMF. A fiber-optic probe accurately measures thermal dosage and automatically maintains it at 43 Celsius for 30 minutes. After the treatment, all cells are gathered, and a number of critical immunological and cytotoxic genetic and protein pathways are likewise elevated by the 8 Gy dosage. The thermotolerance/immunogenic HSP70, chemokines CXCL10, CXCL11, CXCR3, innate immune activators TLR3, TLR4, the MDM2, and mTOR negative regulators of p53, the pro-apoptotic protein PUMA, and the cell death receptor Fas are among the genes most effectively stimulated by the mNPH/radiation combination. Furthermore, protein expression changes verified the genetic alterations, including HSP70, p-mTOR, and p-MDM2. These findings demonstrate that when low dosage mNPH and radiation are employed together, they not only boost the expression of key immunological and cytotoxic molecules but also significantly amplify the impact (27).

#### Fe_3_O_4_

On the genetic level, magnetic nanoparticles can influence the targeting of angiopoietin 2-small interfering RNA (Ang2-siRNA) plasmid/chitosan in melanoma mouse models A-375. 0.15 g magnetic Fe_3_O_4_ nanoparticles are dispersed into 20 ml of 1.5% chitosan. The mouse model is divided into three groups: control, non-targeting, and targeting. The control group is given normal saline, while the non-targeting and targeting groups are given particles *via* the tail vein; the control and targeting groups are exposed to an external magnetic field, while the non-targeting group is not. The mice are killed to confirm the particle distributions in the tumor tissues, and the tumor tissues are stained with hematoxylin, eosin, and Prussian blue. In the tumor tissues, the control group has zero Prussian blue staining, the non-targeted group has moderately positive Prussian blue staining, and the targeting group has significantly positive Prussian blue staining. Under an external magnetic field, Ang2-siRNA plasmid vector/chitosan magnetic nanoparticles go directly towards tumor tissues, demonstrating excellent targeting (28).

#### SCMIOPs

Hydrothermal synthesis yields a novel type of magnetite (Fe_3_O_4_) particles known as “Single-Crystalline Micrometric Iron Oxide Particles” (SCMIOPs). Synthetic control over the single Fe_3_O_4_ phase composition as well as the particle size range of 1 μm to 30 μm is possible. Researchers discover that these particles have vanishing remanent magnetization (σr=0.28 emu/g) and coercive force (Hc=1.5 Oe), indicating superparamagnetic-like behavior (unexpected at micrometric particle size), as well as remarkably high saturation magnetization (σs=95.5 emu/g), ensuring strong magnetic response and lack of agglomeration after the magnetic field is removed. These characteristics make such particles suitable for biomedical applications, since they may be employed instead of magnetic nanoparticles, which have disadvantages such as agglomeration and inadequate magnetic response. In this regard, cytocompatibility/cytotoxicity tests on human cells are performed, and the results clearly indicate that SCMIOPs are cytocompatible for healthy cell lines HaCaT (human keratinocytes) and HEMa (primary epidermal melanocytes) and cytotoxic for neoplastic cell lines A375 (human melanoma) and B164-A5 (murine melanoma) in a dose-dependent manner (29).

#### NPrCAP/magnetite

Melanogenesis substrate N-propionyl-4-S-cysteaminylphenol (NPrCAP) is conjugated with magnetite nanoparticles to create functionalized magnetite nanoparticles, NPrCAP/magnetite. NPrCAP/magnetite nanoparticles (118 kHz, 30.6 kA/m) are injected into B16-F1 melanomas in C57BL/6 mice, which are then treated with hyperthermia using an AMF. When the tumor-draining lymph nodes become larger, T-cell receptor (TCR) repertoire is restricted in tumor-infiltrating lymphocytes and preferred Vβ11^+^ T cell growth is detected. The existence of clonally amplified T lymphocytes is indicated by DNA sequences of the third complementarity-determining regions. These findings suggest that T cells oriented toward a small number of epitopes dominate the T-cell response in B16-F1 melanomas following hyperthermia and that epitope-specific T cells typically employ a limited TCR repertoire (30).

#### Iron-dextran nanoparticles

TCR signaling has been investigated using iron-dextran nanoparticles functionalized with T cell activating proteins. The triggering of membrane receptors by nanoparticles is susceptible to physiologically controlled changes in TCR clustering that occur after T cell activation. Nanoscale artificial antigen-presenting cells (nano-aAPC) bound two times more TCR on activated T cells with clustered TCR than on naïve T cells, resulting in a lower activation threshold. A magnetic field (0.2 T) is utilized to induce aggregation of paramagnetic nano-aAPC to improve T cell activation, resulting in a doubling of TCR cluster size and enhanced T cell proliferation *in vitro* and following adoptive transfer *in vivo*. T cells stimulated by nano-aAPC in a magnetic field repress B16 melanoma growth, demonstrating that this novel approach, which uses magnetic field-enhanced nano-aAPC stimulation to generate large numbers of activated antigen-specific T cells, can generate clinically relevant numbers of activated antigen-specific T cells with clinically relevant applications for adoptive immunotherapy (31).

## The influence of EMF on drug uptake

Despite the impact on the growth and immunity of melanoma, EMF also regulates cell polarity, which allows it to affect drug uptake and medicinal effectiveness. The reviewed chemicals or drugs and EMF’s influence on them are collected in **Table 2**. The detailed effect of each drug is explained below.

**Table 2:** EMF’s impact on drug uptake: listed in alphabetical order.

Drug	Effects	Reference
5-fluorouracil (5-FU)	Under weak external magnetic fields, 5-FU can access the intracellular area of B16-F10 melanoma cells more effectively.	(35)
Camptothecin (CPT)	Exposure to external static magnetic field enhanced uptake of CPT, which inhibited the proliferation of human Me300 melanoma cells.	(36)
Cisplatin (CDDP)	The combination of pulsed electromagnetic field (PEMF) and CDDP hinder the growth of B16-F10 melanoma tumors.	(37)
Curcumin	Employing an alternating magnetic field helps nanogels penetrate the intracellular area of B16-F10 melanoma cells and hence trigger the release of curcumin.	(38)
Doxorubicin (DOX)	Targeted-magnetoliposomes help the transportation of DOX across blood-brain-barrier cell model and enhance the anti-proliferation effect of B16 melanoma cells under a permanent magnetic field.	(39)
Epirubicin (EPI)	Epirubicin can be linked with functionalized superparamagnetic iron-oxide nanoparticles (SPION) to inhibit melanoma WM266 cell proliferation under an external magnetic field.	(40)
Hematoporphyrin monomethyl ether (HMME)	Magnetic nanoparticles help with HMME absorption and enhance photodynamic killing effect in B16-F10 melanoma cells.	(41)
N-propionyl-cysteaminylphenol (NPrCAP)	After conjugating NPrCAP to the surface of magnetite nanoparticles and exposure to alternating magnetic field, the growth of B16 melanoma tumor is suppressed.	(42)
Nile red (NR)	Under alternating magnetic field, the release of NR is accelerated in MEL-5 melanoma cell line.	(43)
Vinblastine	External magnet improves the antitumor effect of vinblastine and the suppression of metastasis in B16-F10 melanoma.	(44)
Zinc phthalocyanine (ZnPc)	The cell vitality of B16-F10 melanoma is significantly reduced after photodynamic and hyperthermia treatments of synthesized magnetoliposomes loaded with ZnPc complexed with cucurbituril.	(45)

### 5-fluorouracil (5-FU)

5-FU’s delivery functions are affected by weak external magnetic fields. Medical diagnosis and therapy could be enabled by remotely optical sensing and drug administration utilizing an environmentally-guided magnetically-driven hybrid nanogel particle. Such multifunctional hybrid nanogels (200 nm) are created by first synthesizing magnetic nickel (Ni) nanoparticles (NPs), then growing fluorescent metallic silver (Ag) on the surface of Ni NPs, and finally covering the Ni-Ag bimetallic NP cores (18 ± 5 nm) with a pH-responsive copolymer gel shell of poly(ethylene glycol-co-methacrylic acid) [p(EG-MAA)]. When the pH-responsive p(EG-MAA) gel shell is added to magnetic and fluorescent Ni-Ag NPs, the polymer-bound Ni-Ag NPs become pH-responsive across the medically relevant range of 5.0-7.4. As the pH value drops from 7.4 to 5.0, the hybrid nanogels may adapt to the surrounding pH and control sensitivity in response to an external magnetic field (0.1 T), resulting in the accumulation of hybrid nanogels between hours to seconds. The pH-dependent magnetic response of the hybrid nanogels is further combined with pH change to fluorescence signal transduction and pH-regulated anti-cancer medication delivery capabilities (a model drug 5-fluorouracil). The hybrid nanogels can penetrate cellular barriers to light up the mouse melanoma B16-F10 cells and access the intracellular area. The capacity to address the complexity of biological systems could be enhanced by the numerous responsive hybrid nanogel that can be controlled in unison with endogenous and external activation (35).

### Camptothecin (CPT)

Tumor-selective magnetically-enhanced drug delivery may be achieved by attaching therapeutic medicines to ultrasmall superparamagnetic iron oxide nanoparticles (USPIOs), which would allow intracellular re-release of the active medication *via* cell-specific mechanisms. To test this theory, the anticancer drug CPT is covalently bonded to biocompatible USPIOs (iron oxide core, 9–10 nm; hydrodynamic diameter, 52 nm). USPIOs are coated with polyvinylalcohol/polyvinylamine (PVA/aminoPVA). CPT is attached to the aminoPVA as a physiologically labile ester substrate for cellular esterases on one end and as an amide on the other end according to a bifunctional, end-differentiated dicarboxylic acid linker. *In vitro*, CPT–USPIO conjugates inhibit the proliferation of human Me300 melanoma cells. Transmission electron microscopy (iron oxide core) and fluorescence microscopy verify the intracellular localization of CPT–USPIOs, indicating that they are found in lipid vesicles, and the use of an external static magnetic field during exposure enhances CPT–USPIO uptake in melanoma cells (36).

### Cisplatin (CDDP)

Similar to traditional electroporation, the pulsed electromagnetic field (PEMF) generates a pulsing electric field, which allegedly enhances membrane permeabilization of the exposed cells. Compared to traditional electroporation, the major benefits of PEMF-mediated electroporation include contactless and painless application, as well as efficient electroporation. Therefore, contactless PEMF is a viable medication delivery method. A magnetic field pulse generator is connected to a round coil applicator to accomplish noninvasive electroporation. Subcutaneous mouse B16-F10 melanoma tumors are treated with CDDP (4 mg/kg) intravenously, PEMF (480 bipolar pulses at 80 Hz, pulse length of 340 μs), or a combination of both treatments (electrochemotherapy - PEMF + CDDP). The anticancer efficacy of CDDP-mediated PEMF electrochemotherapy is equivalent to traditional CDDP-mediated electrochemotherapy, with tumor growth delays of 2.3 days and 3.0 days, respectively. The injection of CDDP alone has no influence on tumor development, as does merely exposing tumors to PEMF. The chemical formula of CDDP is PtCl_2_(NH_3_)_2_. Increased medication absorption into the electroporated tumor cells, as measured by the quantity of platinum (Pt) attached to the DNA, is linked to the anticancer impact of the combination therapy. The amount of Pt taken up by cells increases by almost 2-fold. These results indicate that PEMF-induced electroporation could be used in medicinal applications such as electrochemotherapy (37).

### Curcumin

Multifunctional core-shell hybrid nanogels with fluorescent and magnetic characteristics could be used for simultaneous optical temperature monitoring, tumor cell imaging, and magnetic/near-infrared (NIR) thermally responsive drug (i.e. curcumin) carriers. The bifunctional nanoparticles (BFNPs) are coated with a thermo-responsive poly(N-isopropylacrylamide-co-acrylamide) [poly(NIPAM-AAm)]-based hydrogel. Due to the fluorescent carbon dots embedded in the porous carbon shell, the BFNPs in hybrid nanogels have excellent photoluminescence and photostability as well as drug accumulation potential. BFNPs also have great magnetic–thermal conversion ability because of the superparamagnetic iron oxide nanocrystals clustered in the core. By varying the local temperature of environmental media, the thermo-responsive poly(NIPAM-AAm)-based gel shell can not only manipulate the physicochemical environment of the BFNPs core to control the fluorescence intensity for sensing variations in temperature but also regulate the release rate of the loaded anticancer drug curcumin. Furthermore, the carbon layer of BFNPs may absorb and convert NIR light to heat, resulting in increased release of curcumin and improved therapeutic effectiveness of curcumin-loaded hybrid nanogels. Employing an alternating magnetic field, the superparamagnetic iron oxide nanocrystals in the core of BFNPs can induce localized heating, causing a phase shift in the polymer gel and triggering the release of curcumin. Finally, the multifunctional hybrid nanogels can penetrate cellular barriers to light up the mouse melanoma B16-F10 cells (38).

### Doxorubicin (DOX)

Multifunctional magnetoliposomes (MLs) with active and magnetic targeting capabilities are being tested as drug targeting platform systems. Freeze-drying/extrusion is used to make ultra-small superparamagnetic iron oxide (USPIO)-encapsulating MLs, which are then adorned with one or two ligands for targeted-magnetoliposomes (t-MLs) and actively loaded with DOX. DOX’s loading and retention are not influenced by co-entrapment of USPIOs, and USPIO loading/retention is not regulated by DOX. Ligand attachment yields and DOX loading efficiency are both sufficient, at 78–95% and 89–92%, respectively. The presence of ligands has no effect on DOX or USPIO loading. In contrast to free USPIOs, MLs exhibit high magnetophoretic mobility (MM) that is unaffected by surface coating with polyethylene glycol (PEG, up to 8 mol%), but is slightly reduced by Cholesterol (Chol) incorporation in their membrane or when functional groups are fixed on their surface. The most significant MM-determining factor is ML size, which is directly proportional to the number of USPIOs entrapped per vesicle. When the ML size is increased from 69 to 348 nm, the MM increases by 570%. Through the improvement in both the transportation across blood-brain-barrier cell model and the influence of anti-proliferation on B16 melanoma cells, the targeting potential of t-MLs is justified. Under a permanent magnetic field (Fe concentration 1.2, 2.4, 4.5 ppm), there is a possibility to further improve t-ML targeting efficiency (39).

### Epirubicin (EPI)

Transdermal delivery of chemotherapeutic drugs is a long-standing problem in tumor therapy. Epirubicin (EPI), a model anticancer drug, is linked to functionalized superparamagnetic iron-oxide nanoparticles (SPION). EPI–SPION, a possible drug delivery vector that employs magnetism for targeted transdermal treatment of skin cancers, is created by covalently modifying the SPION. With a saturation magnetism intensity of 77.8 emu/g, the spherical EPI–SPION composite has outstanding magnetic responsiveness. EPI-SPION is also a pH-sensitive drug delivery that targets the acidic microenvironment seen in tumor tissues and endosomes/lysosomes. SPION has high biocompatibility on human keratinocyte HaCaT cells and melanoma WM266 cells. The nanoparticles (i.e. SPION) can decrease WM266 cell growth after being conjugated with EPI; the inhibitory impact on tumor proliferation is dose-dependent. The EPI–SPION composite may penetrate deep within the skin when driven by an external magnetic field, according to *in vitro* transdermal experiments. The magnetic-field-assisted SPION transdermal vector can use follicular routes to bypass the stratum corneum (40).

### Hematoporphyrin monomethyl ether (HMME)

Fullerene and fullerene derivatives have been intensively investigated for applications in biomedicine because of their physical and chemical characteristics. Iron oxide nanoparticles (IONPs) are decorated on the surface of fullerene (C60), and PEGylation is used to improve the solubility and biocompatibility of C60-IONP, resulting in a multifunctional C60-IONP-PEG nanocomposite with strong superparamagnetism and powerful photodynamic therapy capacity. A novel photodynamic anti-cancer medication HMME is coupled to C60-IONP-PEG, resulting in a C60-IONP-PEG/HMME drug delivery system with outstanding magnetic targeting capabilities in cancer therapy. Due to a 23-fold greater HMME absorption of tumor and significant photodynamic activity of C60-IONP-PEG, the photodynamic cancer cell killing effect of C60-IONP-PEG/HMME is observed in both cultivated B16-F10 cells *in vitro* and *in vivo* mouse tumor model (41).

### N-propionyl-cysteaminylphenol (NPrCAP)

NPrCAP, a melanogenesis substrate, is preferentially absorbed into melanoma cells and suppresses their development by releasing cytotoxic free radicals. When exposed to an AMF, magnetite nanoparticles also destroy cancer cells and produce heat shock protein (HSP). By conjugating NPrCAP to the surface of magnetite nanoparticles (NPrCAP/M), one study investigates if a chemo-thermo-immunotherapy (CTI therapy) method for improved melanoma management may be created. Researchers evaluate the viability of this method in B16 mouse melanoma and look at the effects of temperature, frequency, and interval on re-challenged melanoma growth inhibition. The therapy strategy against the initial transplanted tumor, which includes AMF exposure once a day every other day for three treatments, not only stops the main transplant from growing but also prevents the subsequent, re-challenge transplant from growing. Instead of controlling magnetite alone or without AMF exposure, NPrCAP/M with AMF exposure results in the most substantial growth suppression of the re-challenge tumor and extends the survival of tumor-bearing mice. The therapeutic impact of heat is stronger at 43 Celsius than at 41 Celsius or 46 Celsius, and HSP70 production is highest at 43 Celsius, compared to 41 Celsius and 46 Celsius. The re-challenged melanoma transplant site is infiltrated with CD8^+^ T cells (42).

### Nile red (NR)

Reversibly crosslinked **(**RCL) nanogels composed of thermo-responsive poly(vinyl alcohol)-b-poly(N-vinylcaprolactam) copolymers are coupled with maghemite nanoparticles to provide novel drug delivery systems (DDS). The surface-functionalized superparamagnetic maghemite nanoparticles are used to create the crosslinking, which give the DDS thermo-, pH-, and glucose-responsive properties. The hydrophobic drug NR can be loaded easily and well trapped within the resulting RCL nanogels when pH is 7.4. Under alternating magnetic field (AMF, 755 kHz, 14 mT), the release of NR is accelerated, with mild heating of the surrounding. Cytotoxicity of RCL nanogels against MEL-5 melanoma cell line reveals sufficient biocompatibility for biomedical application (43).

### Vinblastine

Magnetic drug targeting (MDT) is a well-studied strategy for selectively delivering chemotherapeutic medicines to tumor cells; nevertheless, targeting tumor endothelial cells using this way is a relatively recent concept. Although positively charged (cationic) liposomes have a very high affinity for tumor vasculature, heterogeneous targeting is common. The use of an externally applied magnetic field is examined in conjunction with magnetic cationic liposomes (MCLs) for cancer treatment in order to enhance the overall effectiveness of targeting tumor arteries. In addition, the anticancer effect of vinblastine loaded in MCLs is investigated using B16-F10 murine model of melanoma. When compared to untreated and free drug control groups, the administration of vinblastine-loaded MCLs with the magnet have a substantial anticancer impact, lowering the occurrence of tumor nodules at preferred regions of metastasis. For the vinblastine-loaded MCL groups, cluster of differentiation 31 (CD31) immunostaining indicates reduction in the overall length of tumor blood vessels, altered vascular architecture, and discontinuities in the tumor vascular lining (44).

### Zinc phthalocyanine (ZnPc)

A study synthesizes magnetoliposomes (MLs) loaded with zinc phthalocyanine (ZnPc) complexed with cucurbituril (CB) (CB : ZnPc-MLs) and evaluates their *in vitro* photodynamic (PD) and/or hyperthermia (HT) effects using B16-F10 melanoma cells. The average diameter and zeta potential of the liposomal formulations are measured: the typical vesicle size is between 150 and 200 nm, and the polydispersity index (PdI) is between 0.093 and 0.230. With values ranging from 48 to 57 mV, the zeta potential is considerably positive. The colorimetric MTT technique is used to measure cell vitality (CV) following PD and HT treatments. Melanoma cells are first treated with the liposome formulation without the use of light or a magnetic field, and cell survival is shown to be comparable to that of control cells (p > 0.05). Separate photodynamic and hyperthermia tests are used to show that PD is more efficient than HT in lowering the CV of neoplastic cells. The CV of B16-F10 cells is reduced considerably more effectively when both PD and HT treatments are used together. The CV is roughly half that of PD applied alone at the maximum light dosage (2 J/cm^2^) and under magnetic field activation. As a result, the utilization of a photosensitizer-loaded magnetoliposome for combination photodynamic therapy (PDT) and magnetohyperthermia (MHT) treatment of malignant melanoma has great potential for future medical treatments (45).

## The influence of EMF on clinical therapies

**Figure 1:**
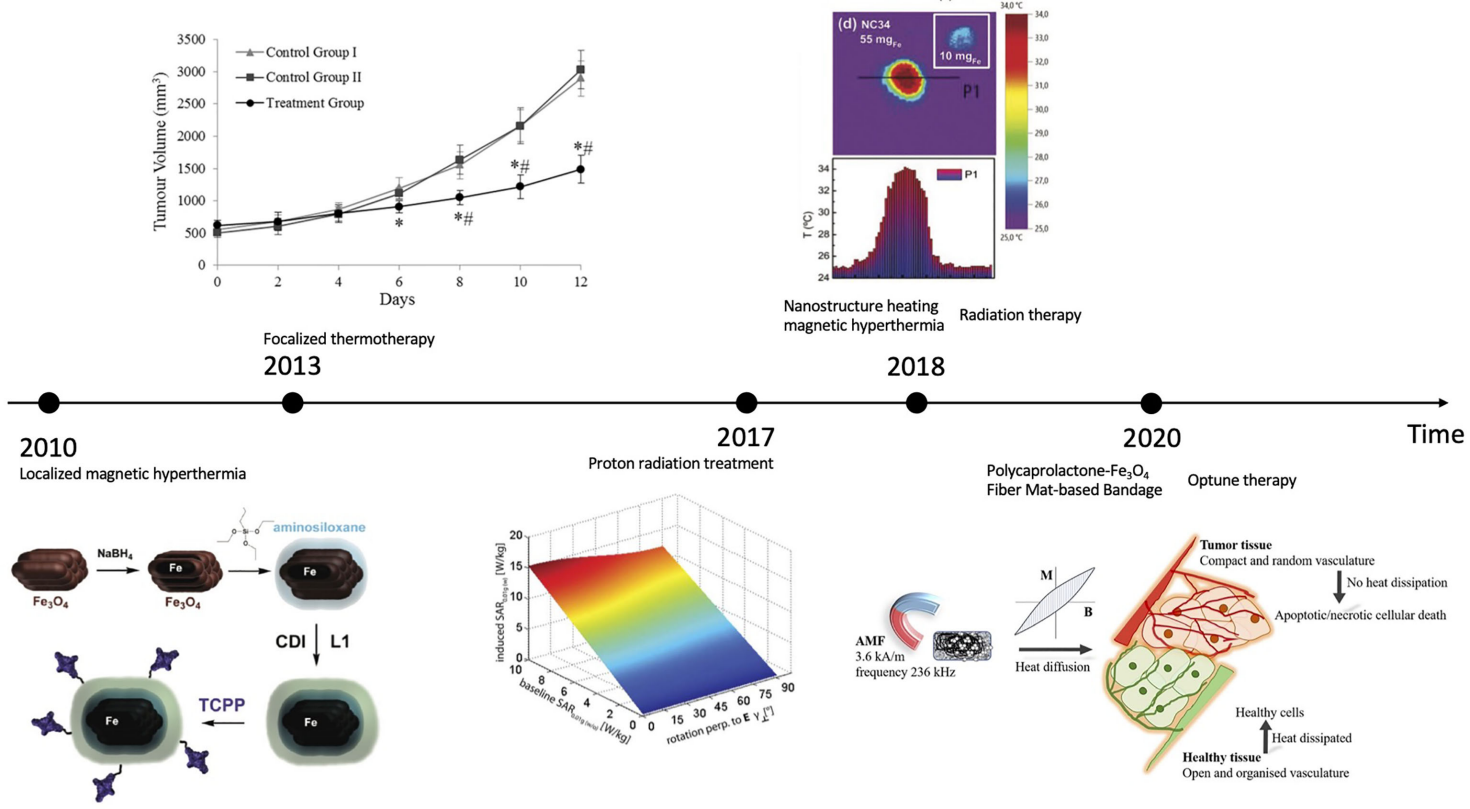
Schematic representation of EMF’s influence on clinical therapies: a chronological comparison (2010-2020). CREDIT: Bibliography 46–52. For (46): Reprinted (adapted) with permission from {Rachakatla RS, Balivada S, Seo G-M, Myers CB, Wang H, Samarakoon TN, et al. Attenuation of mouse melanoma by A/C magnetic field after delivery of bi- magnetic nanoparticles by neural progenitor cells. ACS nano. (2010) 4(12):7093– 104. doi: 10.1021/nn100870z}. Copyright {2010} American Chemical Society

Despite drug uptake, EMF also has notable impact on clinical therapies. Below we collect various types of clinical therapies influenced by EMF in the sequence of published time. In **Figure 1**, we also created a schematic representation of EMF’s influence on clinical therapies in chronological order.

### Localized magnetic hyperthermia (2010)

The possibility of using localized magnetic hyperthermia as a cancer therapy has resurfaced, especially if it can be tailored to the tumor location. In a mouse model of melanoma, whether tumor-tropic neural progenitor cells (NPCs) can be used as cell delivery vehicles to achieve preferential accumulation of core/shell iron/iron oxide magnetic nanoparticles (MNPs) is investigated. MNPs are created with aminosiloxane porphyrin functionalized aminosiloxane porphyrin; their efficiency on cell survival and loading are tested, and neural progenitor cells are transplanted with them into melanoma mice. NPCs are loaded with core/shell Fe/Fe_3_O_4_ MNPs effectively and with minimum cytotoxicity; the MNPs accumulate in the cytosol as NPC aggregates. The NPCs loaded with MNPs are able to travel to subcutaneous melanomas, and the targeted administration of MNPs results in significant tumor shrinkage following AMF exposure. The tumor decreases in a short period (24 hours) following the final of three AMF treatments (46).

### Focalized thermotherapy (2013)

Strong EMF helps with highly focalized thermotherapy (HFT) using a ferrimagnetic cement (FC) in the treatment of a B16-F10 melanoma mouse model by low-temperature hyperthermia. The magnetic vehicle for HFT is FC, which is injected into the tumor. Radiography is used to determine the position of the FC within the tumor, and a thermal camera is used to measure its capacity to create heat when subjected to an external high-frequency magnetic field (HFMF, 10kHz). The HFT therapy consists of three HFMF treatments separated by 48 hours, each lasting 30 minutes and increasing the tumor temperature by 5–6 degrees Celsius. FC samples are characterized using scanning electron microscopy (SEM) and energy dispersion spectroscopy at the end of the experiment (EDS). The localized temperature of tumor increase is confirmed by the thermal camera. HFT treatments reduce tumor growth by approximately 70% compared to controls, which is attributed to cell necrosis and apoptosis (48).

### Proton radiation treatment (2017)

Uveal melanoma is commonly treated with proton radiation treatment (PRT). Ocular tantalum markers (OTMs) are implanted in PRT patients for treatment planning. For 3D tumor imaging and PRT planning, ultra-high-field magnetic resonance imaging (MRI) is a promising approach. The safety and compatibility of OTMs in magnetic resonance (MR) at 7.0 Tesla are investigated. Deflection angle measurements (DAMs), EMF simulations for estimating specific absorption rate (SAR), and temperature simulations for assessing radiofrequency heating utilizing a bow-tie dipole antenna for transmission are all part of the MR safety evaluation. Susceptibility artifacts in agarose, *ex vivo* pig eyes, and an *ex vivo* tumor eye are used to test MR compatibility using gradient echo and rapid spin-echo imaging. DAM (α < 1°) reveals no danger from magnetically induced OTM deflection. EMF simulations illustrate that an OTM can be approximated by a disk, hence demonstrating the need for averaging masses of m_ave_ = 0.01 g to adapt for the OTM, provided that SAR_0.01g,max_=2.64 W/kg (P_in_=1W) in OTM presence. A transfer function is developed, enabling SAR_0.01g_ approximation for specific patient circumstances without the OTM. Thermal simulations expose a minor OTM-related temperature increase (dT<15 mK). Susceptibility artifact size (<8mm) and location suggest no restrictions for MRI of the nervus opticus (49).

### Nanostructure heating magnetic hyperthermia (2018)

Magnetic hyperthermia is an oncological treatment in which magnetic nanostructures serve as heat transducers in a radiofrequency field, raising tumor temperature and destroying malignant cells. The effectiveness of nanostructure heating is determined by the field parameters as well as the nanostructure characteristics and movement inside the tumor. Such nanostructures are frequently bench-marketed improperly in the colloidal form and with field settings that are far from the therapeutic values. The nanoclusters prepared are composed of iron oxide magnetite nanoparticles aligned crystallographically. The specific absorption rate (SAR) values of the nanoclusters are measured calorimetrically in physiological fluids, agarose-gel-phantoms and *ex vivo* tumors extracted from mice with B16-F10 melanoma cells. The findings are thoroughly examined in terms of nanoclusters’ structural and magnetic characteristics utilizing a portable, multifunctional applicator with medical field settings of 100 kHz and 9.3 kA m^-1^. The SAR values of fluid suspensions or agarose-gel-phantoms are visibly insufficient to forecast the true tissue temperature rise, or the dose required to heat a tumor, according to a detailed examination of the nanoclusters’ heating capability in the three milieus. In addition to nanostructure mobility, perfusion, and local thermoregulation, the nanostructure distribution inside the tumor is important for efficient heating. There is a reduction in the effective heating efficiency of magnetic material inside the tumor. To obtain the necessary temperature increase, the dose must be raised significantly from the SAR values anticipated from fluid or agarose (50).

### Radiation therapy (2018)

Magnetic nanoparticles aid radiation therapy (RT) in the treatment of canine oral melanoma. An alternating magnetic field is used to activate a plant-based virus-like nanoparticle (VLP) and a 110 nm diameter magnetic iron oxide nanoparticle (mNPH) to create moderate heat (43°C/60 min). The RT is used alone or in conjunction with one or both adjuvants. During the RT regimen, the VLP (4x200 g) and mNPH (2x7.5 mg/gram tumor) are administered intratumorally. The tumors are assessed immunohistopathologically before and 14-21 days after therapy. The addition of mNPH to a hypofractionated radiation regimen improves immune cell infiltration in the tumor, increases tumor control intervals, and has significant systemic therapeutic potential (51).

### Polycaprolactone-Fe_3_O_4_ fiber mat-based bandage (2020)

EMF electrospinning process is used to create Fe_3_O_4_ nanoparticles using polycaprolactone (PCL) fiber-based bandages. The bandage’s effectiveness is examined in parental/doxorubicin hydrochloride (DOX)-resistant HeLa cells *in vitro*, and in a BALB/c mouse model in the presence of an external alternating current (AC) magnetic field *in vivo*. On the application of an external AMF, the PCL-Fe_3_O_4_ fiber mat-based bandages release heat energy locally and elevate the surrounding temperature in a controlled fashion up to 45 Celsius in a few minutes. The *in vitro* investigation indicates that the increased temperature may considerably kill parental and DOX-resistant HeLa cells. When cells are treated with a DOX-containing fibrous mat in the presence of AMF for 10 minutes, more than 85% of parental HeLa cells are dead and the activity of DOX increases at higher temperatures. After five 15-minute hyperthermic treatments, chemically generated skin cancers in BALB/c mice recover completely within a month (52).

### Optune therapy (2020)

Optune system is one FDA-approved medical device that supports immunotherapy in melanoma patients with brain metastasis. Optune system, also named NovoTTF-200A Therapy, is a battery-powered device that utilizes surface transducer arrays to create Tumor Treating Fields (TTFields) within the human body. Since TTField is a type of alternating electric field, it interferes with cell division and induces tumor regression. Besides, normal brain cells are not affected because alternating electric field has little effect on cells that are not dividing. This enhances the safety of using optune system to treat melanoma (47).

As the time being, clinical therapies related to EMF are becoming more accurate, safe, and comfortable for patients. However, some regulatory challenges could still be improved in the future. First, the targeting of tumor could be more flexible with the process of therapeutic procedures. Since tumor is constantly vibrating on a molecular level and changing its size during the therapy, EMF should be more focused or dispersed accordingly. Second, synergies of multiple EMFs can be explored. EMFs of different frequencies have the potential to better enhance the inhibition of melanoma than one type of EMF alone. Third, the intensity of EMF exposure could be made more individualized for each patient. Therapies should take into consideration of the patient’s age, gender, weight, and past medical history to determine how much EMF to use and how long the exposure should be. In addition, some people are allergic to radiation emitted from electronics. Therefore, before every therapy it would be necessary to ensure the patient does not have electromagnetic hypersensitivity.

## Conclusion

In conclusion, EMFs have both pathological impacts and medical applications on melanoma. In general, the specific frequencies and intensities of EMFs mentioned in this review inhibit melanoma growth for various melanoma cell lines. Weak EMFs reduce melanoma with constant exposure time and alter cellular immunological functioning in melanoma cells. Strong EMFs detach melanoma cell membrane and induce apoptosis of melanoma cells in a much shorter time period. Alternating EMF has multiple synergetic effects on melanoma with magnetic hyperthermia and aids the reduction of tumor. Magnetic nanoparticles better target cytotoxic molecules and intracellular plasmid in melanoma cells and improve the precision of EMF-melanoma experiments.

EMF also promotes drug uptake. Under external EMF, 5-FU, CPT, Curcumin, DOX, and HMME can penetrate the membrane of melanoma cells more effectively. EMF also helps suppress the growth of melanoma proliferation with CDDP, EPI, NPrCAP, Vinblastine, and ZnPc. In addition, NR is accelerated in melanoma cells under alternating EMF.

Furthermore, EMF aids clinical therapies and has progressed greatly in the past decade. EMF decreases the procedure time for localized magnetic hyperthermia and nanostructure heating magnetic hyperthermia, as well as reducing tumor growth for focalized thermotherapy and optune therapy. Magnetic resonance better targets uveal melanoma in proton radiation treatment. EMF increases tumor control intervals in radiation therapy when treating canine oral melanoma. EMF electrospinning process is also used to create Fe_3_O_4_ nanoparticles using polycaprolactone (PCL) fiber-based bandages.

Above all, EMF not only influences the development of melanoma but also has a notable impact on the uptake of the anti-cancer drug for melanoma and clinical therapies for melanoma. It is foreseeable that in the future, there will be more research about how EMF can be utilized to understand and treat melanoma to enhance the efficiency and accuracy of medications.

## Author contributions

YD is the first author who did most of the writing. YK is the corresponding author who instructed the research. XW, ZG, and QG helped with brainstorming and editing. All authors contributed to the article and approved the submitted version.

## Funding

Grant No. 81972557 from National Natural Science Foundation of China, Beijing Natural Science Foundation (7202022),National Key R&D Program of China (2019YFA0904400),No. Z191100006619006 from the Beijing Municipal Science and Technology Commission.

## Conflict of interest

The authors declare that the research was conducted in the absence of any commercial or financial relationships that could be construed as a potential conflict of interest.

## Publisher’s note

All claims expressed in this article are solely those of the authors and do not necessarily represent those of their affiliated organizations, or those of the publisher, the editors and the reviewers. Any product that may be evaluated in this article, or claim that may be made by its manufacturer, is not guaranteed or endorsed by the publisher.
